# When Addressing Trauma Makes a Difference: A Case Report of Undiagnosed Complex Post-traumatic Stress Disorder

**DOI:** 10.7759/cureus.51640

**Published:** 2024-01-04

**Authors:** Mauro Pinho, Daniela O Martins, Mónica F Santos, Francisco Coutinho

**Affiliations:** 1 Acute Psychiatry Service Unit, Centro Hospitalar Universitário de Santo António - Hospital de Magalhães Lemos, Porto, PRT

**Keywords:** major depressive disorder, childhood sexual abuse, trauma, cognitive-behavioral therapy, personality disorder, bipolar disorder, complex post-traumatic stress disorder

## Abstract

Complex post-traumatic stress disorder (C-PTSD) is an emergent diagnosis, which acknowledges the impact of prolonged interpersonal abuse on affect regulation, interpersonal functioning, and self-concept. We present the case of a 59-year-old woman who remained undiagnosed and untreated for this condition for three decades while under follow-up in mental health services for the diagnosis of personality disorder and bipolar disorder. The patient suffered repeated sexual abuse in her childhood, resulting in intrusive traumatic memories she emotionally and cognitively avoided, dissociative amnesia, a persistent inability to experience positive emotions, a persistent sense of guilt, re-experiencing phenomena, and hypervigilance toward others and their intentions to harm her. She persistently believed herself to be worthless, defective, inferior, and lacking value; had a history of affective dysregulation resulting in suspicion of bipolar disorder; and displayed a pattern of relationship avoidance. Addressing chronic trauma and assessing its impact offered deeper contextualization of the patient’s symptoms and proved pivotal in redefining her diagnosis and providing access to trauma-focused psychotherapy, which is the mainstay of treatment for C-PTSD.

## Introduction

Acknowledging the complex psychological sequelae that result from prolonged interpersonal abuse, the 11th edition of the International Classification of Diseases, released in 2018, recognizes complex post-traumatic stress disorder (C-PTSD) as a new diagnosis, distinct from post-traumatic stress disorder (PTSD) [1].

The diagnosis of C-PTSD includes the three core elements of PTSD, namely, re-experiencing traumatic events, avoidance of stimuli associated with these events, and a persistent sense of threat. Additionally, C-PTSD comprises three other symptom clusters, representing chronic disturbances in self-organization, namely, a negative self-concept, emotional dysregulation, and interpersonal dysfunction [2].

Typically, C-PTSD results from chronic trauma related to events such as repeated physical or sexual abuse, domestic violence, prolonged combat, torture, or genocide. However, an individual who has experienced repeated sexual abuse in childhood can develop PTSD, rather than C-PTSD, if they have protective factors, such as adequate caregivers or good social support. The reverse also applies, individuals exposed to a single traumatic event may develop C-PTSD if they have a poor social support network or a history of other adverse childhood events [2].

It is estimated that the one-month prevalence of C-PTSD ranges from 1% to 8% in the general population [2]. In at-risk populations, however, this prevalence appears to be much higher, ranging from 16% to 38% among refugees seeking treatment [3], 18% among British firefighters [4], and 13% among American war veterans [5]. The evidence supporting the diagnosis of C-PTSD is strong and has been replicated in multiple countries and cultures [6]. C-PTSD is an emerging, prevalent, and clinically relevant nosological entity that is more debilitating than PTSD [7].

The most relevant differential diagnosis for C-PTSD is borderline personality disorder (BPD). In BPD, however, interpersonal relationships are intense and unstable, whereas in C-PTSD, they are avoided. In BPD, the sense of identity is diffuse and unstable, whereas in C-PTSD, the self-concept is persistently negative. In addition, impulsivity and suicidal and parasuicidal behaviors are more common in BPD. Although BPD is often associated with trauma, it should also be emphasized that traumatic events are not necessary for the diagnosis of this condition [8].

We present a case report of a patient with C-PTSD who remained undiagnosed and untreated for three decades while under follow-up in mental health services for the diagnosis of personality disorder and bipolar disorder.

## Case presentation

Our patient is a 59-year-old Portuguese female, who is divorced, living with her brother, her son, and his girlfriend, while working a full-time job as a medical secretary.

After the birth of her only son, at the age of 27, the patient started to feel a low mood, irritability, and a loss of interest in her activities, having been referred for an outpatient psychiatric consultation and being diagnosed with postpartum depression. She was treated with antidepressants, benefitting from a partial response.

At the age of 36, the patient began to feel irritable, with a low mood, sleeping more than usual, lacking interest or pleasure in her usual activities, spending much time in bed, and presenting nonsuicidal self-injury (cuts in her forearms). She believed these symptoms were triggered by an abusive relationship with a partner, who managed to seduce her, gaslight her, and take charge of her finances and property. Her symptoms fluctuated, with periods of agitation, impulsivity, and reckless behavior. She claimed her partner successfully manipulated her into selling her apartment and writing a will, claiming it was her intent that he got custody of her son and inherited all her property. In her will, she also expressed her last wishes, as she intended to commit suicide. She got agitated and violent against her son and partner as well, so authorities were called and she was taken to the psychiatry emergency room. Her partner claimed she was being treated with valproate semisodium and olanzapine, but the patient accused him of drugging her with these prescriptions to manipulate and control her. She also believed her family wanted to take her children from her. She was diagnosed with major depressive disorder featuring psychotic symptoms and was involuntarily admitted as an inpatient to a psychiatric unit. During hospitalization, her mood was described as unstable; however, she never met the full criteria for a manic, mixed, or hypomanic episode and was still diagnosed with bipolar disorder, at the time. During her follow-up, her affective dysregulation, interpersonal difficulties, and periods of suicidal ideation became more evident, so her diagnosis was changed to a personality disorder with paranoid and histrionic features.

The patient remained stable until three years later, when she presented with another period of low mood and suicidal ideation, being admitted as an inpatient and getting discharged the next day.

There was no record of any other major mood episode, since then, even for seven years wherein the patient abandoned her psychiatric medication. Four years ago, the patient maintained some periods of mood lability and sadness, initiating treatment with lamotrigine 200 mg per day. She had no recurrence of major depressive or manic episodes until today. She had not presented other self-injurious behavior.

We have been in charge of the patient’s consultation since May 2022. On psychiatry records, she was described as “very difficult,” paranoid, hostile and defensive, avoiding talking about her past, and almost having been discharged because of lack of cooperation. Later, she reported that, from the age of six to 11 years, she experienced repeated sexual abuse by one of her two brothers, who was five years older. She added that the “past should stay in the past, we don’t need to dig it up.” She claimed her biggest goal in therapy was to “stop crying because it is a waste of time” and avoided talking about her traumatic experiences. Whenever we addressed her history of sexual abuse, she would start crying, overtly dysregulated. She learned to dissociate during sexual abuse and dissociated while recalling her experience: “the trick is simple - to talk about it like if I was watching a movie, not participating in it.” She revealed dissociative amnesia regarding part of the traumatic events. She recognized her lack of ability to experience pleasure or positive emotions, explaining “this is the price to pay, but, at least, I don’t also feel bad … I feel never too well, never too bad.” The patient revealed a persistent sense of guilt over her traumatic experiences and avoided distressing memories and reminders of the past while still being troubled by frequent intrusive memories of the events. Her locus of control was internal, and she tended to minimize her suffering.

Her first suicide attempt was at nine years old after hearing her mother claim she could not divorce because of her children. Sexual abuse was also occurring during this period. She recalled being overwhelmed by feelings of guilt, thinking her mother’s life would be better without her. She attempted suicide by taking her father’s psychiatric medication but survived with no medical assistance. She did not know her father’s psychiatric diagnosis, but described him as a man who, at times, “talked with rhymes” (possible flight of ideas), and other times had “an empty stare.” Her father was hospitalized in a psychiatry department and subject to electroconvulsive therapy; thus, we suspect he suffered from a severe mood disorder.

Her family was described as emotionally depriving. At the age of 17, she told her parents about the abuse, but they discredited her claims. She grew up believing she was “wrong, worthless” and that others could not be trusted: “for me, everyone is guilty, until proven innocent … people are always up to something and will, eventually, reveal their true intentions.”

Her son, now 31 years old, reported that the patient’s abusive partner described earlier was, in fact, a criminal, got her mother involved in scams, and was even imprisoned. Her mother truly lost custody of him because of submissive involvement in these crimes, so the patient’s claims were not delusional, as interpreted at the time of her involuntary admission.

After ending her relationship with this partner, the patient avoided new relationships and remained single for at least 15 years. Then, she entered into a relationship with a man, who she described as a wonderful man whom she did not deserve. Even though they have been together for the last five years and rarely fight, they rarely see each other, have never been in each other’s houses, and do not know each other’s families, and the patient admits feeling safer this way: “there’s no need to get too involved … I think things should evolve at a slow pace.” She acknowledges she is afraid to trust her boyfriend, prefers not to get “too caught up in feelings,” and believes she will be, eventually, abandoned and deceived by everyone: “I know how people are and act on the premise that they’ll always going to lie or betray me.” She has no close friends and believes that, even her son, can’t be fully trusted.

The patient had a medical history of asthma, obesity, and hypertension. She denied any history of substance use. Her family history of mental illness included no other relatives besides her father. She was being treated with lamotrigine 200 mg per day, perindopril/indapamide/amlodipine 5/1.25/5 mg per day, and tiotropium bromide 2.5 µg per day.

The patient was diagnosed with C-PTSD and referred to trauma-focused cognitive-behavioral therapy. Since initiating psychotherapy, she became more compliant with psychopharmacological treatment, starting add-on therapy with sertraline 50 mg per day.

## Discussion

Although the patient was previously diagnosed with bipolar disorder and personality disorder, her current C-PTSD diagnosis explains all her psychiatric history and seems the most comprehensive one, acknowledging her history of trauma and informing a comprehensive psychotherapeutic intervention. The patient also had a history of recurrent major depressive disorder, a common comorbidity for C-PTSD [9].

We argue that her diagnosis of bipolar disorder is not likely, as she never experienced clear hypomanic or manic episodes, remained in remission of major depressive episodes since the age of 39, and did not relapse without maintenance therapy.

Regarding her previous diagnosis of personality disorder, we agree that her current symptoms would be compatible with the diagnosis of paranoid personality disorder, even though her clinical picture, in 2001, shared features with BPD.

Nevertheless, C-PTSD explains all the patient’s symptoms and difficulties more comprehensively. The patient suffered repeated sexual abuse in her childhood, resulting in intrusive traumatic memories she emotionally and cognitively avoids, dissociative amnesia, a persistent inability to experience positive emotions, a persistent sense of guilt, re-experiencing phenomena, and hypervigilance toward others and their intentions to harm her. She persistently believes herself to be worthless, defective, inferior, and lacking value; has a history of affective dysregulation resulting in suspicion of bipolar disorder; and experiences severe interpersonal difficulties. She displays a pattern of relationship avoidance, having difficulty being intimate and close to romantic partners and being currently in a relationship with an unavailable and distant man. When she was retraumatized by an abusive partner, she experienced marked alterations in arousal and reactivity, evidencing violent, reckless, and self-destructive behavior.

The treatment of C-PTSD is still poorly understood, given the recent generalized acknowledgment of this entity. Experts recommend therapies similar to those applied to PTSD, namely, trauma-focused psychotherapy, although they admit that a greater number of sessions may be necessary. There is evidence for the superiority of multicomponent psychotherapies, especially those that include emotional regulation strategies, distress tolerance skills, cognitive restructuring, and self-compassion [10]. Dialectical behavioral therapy encompasses such components and yields large effect sizes in the treatment of this pathology [11].

Antidepressant drugs, such as selective serotonin reuptake inhibitors or venlafaxine, are less effective than psychotherapy and should not be used as a standalone treatment. However, pharmacological treatment can contribute to the patient’s clinical stabilization, thus facilitating adherence to psychotherapeutic intervention [2,10].

## Conclusions

Addressing chronic trauma and assessing its impact offered deeper contextualization of the patient’s symptoms and proved pivotal in redefining her diagnosis and informing proper therapeutic intervention. This case report highlights how trauma may disturb interpersonal relationships, affect regulation, and self-concept, resulting in affective symptoms and personality traits that should be understood in the context of the patient’s personal history. Missing these links deters the therapeutic relationship, prevents comprehensive case formulation, and restricts access to the mainstay of treatment, which is trauma-focused psychotherapy.
